# Healthy dietary intake diminishes the effect of cerebral small vessel disease on cognitive performance in older adults

**DOI:** 10.3389/fneur.2025.1508148

**Published:** 2025-03-06

**Authors:** Christopher E. Bauer, Valentinos Zachariou, Colleen Pappas, Pauline Maillard, Charles DeCarli, Arvind Caprihan, Brian T. Gold

**Affiliations:** ^1^Departments of Neuroscience, University of Kentucky, Lexington, KY, United States; ^2^Behavioral Science, University of Kentucky, Lexington, KY, United States; ^3^Departments of Neurology, University of California, Davis, Davis, CA, United States; ^4^Center for Neurosciences, University of California, Davis, Davis, CA, United States; ^5^The Mind Research Network, Albuquerque, NM, United States; ^6^Sanders-Brown Center on Aging, University of Kentucky, Lexington, KY, United States; ^7^Magnetic Resonance Imaging and Spectroscopy Center, University of Kentucky, Lexington, KY, United States

**Keywords:** cerebral small vessel disease, cerebrovascular disease, vascular contributions to cognitive impairment and dementia, peak width of skeletonized mean diffusivity, Mediterranean diet, cognitive reserve, nutrition, free water

## Abstract

**Introduction:**

We evaluated whether regular dietary intake of nutrients commonly found in fish, unsaturated oils, and nuts would moderate the associations between neuroimaging biomarkers of cerebral small vessel disease (cSVD) and cognitive function in older adults.

**Methods:**

Dietary information, Montreal Cognitive Assessment (MoCA) scores, and magnetic resonance imaging (MRI) scans were collected from 71 older adults without dementia (60–86 years). MRI biomarkers of cSVD were calculated for each participant. Multivariate linear regression models were computed using dietary intake as the moderating variable. Covariates included age, sex, and estimated intracranial volume.

**Results:**

Dietary intake moderated the association between several cSVD biomarkers and MoCA scores such that the expected negative association between cSVD biomarkers and cognition was seen at low levels of healthy dietary intake, but not at medium or high levels. A dietary intake by age moderation was not observed.

**Discussion:**

Our findings indicate that healthy dietary intake may confer cognitive reserve against cSVD in older adults.

## 1 Introduction

Vascular contributions to cognitive impairment and dementia (VCID) are very common in older adults and represent a growing public health risk (1–3). VCID commonly results from cerebral small vessel disease (cSVD), which can be assessed *in vivo* using magnetic resonance imaging (MRI), including several recently developed and validated MRI biomarkers (4). It is known that cSVD can increase significantly with age and contributes to cognitive impairment years prior to the development of dementia (2, 5). Risk factors for cSVD include hypertension, hyperlipidemia, unhealthy diet, sedentary lifestyle, and smoking, among others (2, 5).

Despite the knowledge of risk factors, relatively little is known about how modifiable lifestyle variables confer reserve against cSVD and/or its effects on cognition. According to recent consensus frameworks, the term “reserve” serves as an umbrella that can take three specific forms: cognitive reserve, brain reserve, and brain maintenance (6, 7). Cognitive reserve (sometimes called “resilience”) refers to cases in which one’s cognitive function is better than expected by their brain health. Brain reserve refers to cases in which having more brain resources (e.g., larger brains or less cSVD, leaving more viable brain tissue available) is associated with better cognitive function. Finally, brain maintenance (sometimes called “resistance”) refers to less decline in neural resources than expected by one’s age, that is associated with better cognitive performance (6–8).

Brain reserve, cognitive reserve, and brain maintenance have all been studied extensively in the context of Alzheimer’s disease (AD) pathology (6, 7, 9). In contrast, only a handful of studies have explored potential reserve variables in cSVD (10–15). However, findings from this research have been mixed, with some studies finding evidence of cognitive reserve (13, 15–18) or brain maintenance (11), and others reporting null effects [cognitive reserve; (10, 14); brain maintenance (12, 15)].

Several factors could contribute to these discrepant findings. First, the reserve variables explored in these studies (early life education, occupational attainment, leisure activities, etc.) are selected based on evidence of reserve typically seen in the context of AD pathology (10, 13), yet the sensitivity of these factors to reserve against cSVD is relatively unknown. Several of these factors are also considered “static,” or not modifiable at an advanced age (early life education, occupational attainment). Further, most studies report results from only one reserve factor such as education or leisure activities. Additional research is needed to explore multiple reserve factors in the same study participants to assess the specificity of potential factors providing reserve against cSVD.

Dietary patterns are an important but underexplored potential reserve variable against cSVD given the known association between dietary intake and cSVD generally (19–22). The Mediterranean diet (23) in particular has been identified as a potential candidate for cSVD prevention (19, 20, 24, 25). The Mediterranean diet is also strongly associated with improved cognitive performance and reduced risk of cognitive impairment and risk of dementia (26, 27).

These findings warrant the investigation of dietary intake, particularly dietary intake containing core components of the Mediterranean diet, as a factor that potentially builds reserve against cSVD. Here we explored this possibility using a cross-sectional design. While reserve is optimally measured longitudinally, cross-sectional studies remain important, particularly in emerging fields such as this, in that they can provide preliminary data to develop hypotheses for more expensive and time consuming longitudinal studies and aid in formulation of conceptual frameworks (9). We investigated whether dietary factors might contribute to brain reserve, cognitive reserve, or brain maintenance using cross-sectional analyses recommended by the consensus criteria for each type of reserve (6, 7).

Specifically, evidence for brain reserve would come from a main effect of cSVD on cognitive function (i.e., having less cSVD results in better cognitive performance). Evidence of cognitive reserve would come from a statistical interaction between dietary intake and cSVD on cognitive function. For example, older adults with high cSVD may perform better than expected by their brain status if they practice healthy dietary intake, possibly due to molecular, cellular or network alterations. Finally, evidence of brain maintenance (i.e., resistance) would come from an interaction between dietary intake and chronological age on cSVD biomarkers (i.e., older adults show less cSVD than expected by their age if they practice healthy dietary intake).

As our interest here relates to cSVD, we used three extensively validated MRI biomarkers of cSVD from the MarkVCID consortium [white matter hyperintensity volume (WMH volume) (28), free water (FW) (28, 29), peak width of skeletonized mean diffusivity (PSMD) (28, 30)]. Cognition was assessed using a validated measure of cognitive status, the Montreal Cognitive Assessment (MoCA), a test routinely used in clinical settings due to its sensitivity to detect early cognitive dysfunction (31). Finally, we assessed the specificity of our findings related to healthy dietary intake by testing for the presence of similar relationships when using the Cognitive Reserve Index questionnaire (CRIq) (32) as the potential reserve variable.

## 2 Materials and methods

### 2.1 Participants

Seventy-one participants were recruited from the Sanders-Brown Center on Aging (SBCoA) longitudinal cohort (33) and the greater Lexington community. Inclusion criteria for enrollment in the SBCoA longitudinal cohort are neurological and cognitive normality at enrollment (examination based on clinical consensus), willingness to undergo annual physical, neurological, and cognitive examinations, blood draw, appointment of a designated informant for structured interviews, and a minimum of 60 years of age. Exclusion criteria are major psychiatric illness, untreated depression, current substance abuse, medical illnesses that are nonstable, impairing, or affect the CNS, chronic infectious diseases, stroke or transient ischemic attack, epilepsy, meningitis, encephalitis, or a history of head injury. Participants recruited from the community had the same exclusion criteria.

Additional exclusion criteria for the current study were MRI-related contraindications (i.e., claustrophobia, metal implants, metal fragments, pacemakers), brain abnormalities discovered through imaging, or disease affecting the blood (heart disease, kidney disease, anemia). Additional inclusion criteria for the current study was the absence of dementia at the time of the MRI scan, the completion of a nutrient questionnaire [“Newly Developed Antioxidant Nutrient Questionnaire” (NDANQ) (34)], the Cognitive Reserve Index questionnaire (CRIq) (32) and the Montreal Cognitive Assessment (MoCA) (31). Absence of dementia was determined by annual clinical consensus diagnosis or a score of 18 or greater on the MoCA (31), where 18 is the lower cutoff value for mild cognitive impairment.^1^ The MoCA was completed for each participant within 13 months of the scan date.

A total of 71 older adults (ages 60–86) met initial eligibility criteria for the present study. All participants provided informed consent under a protocol approved by the Institutional Review Board of the University of Kentucky.

### 2.2 Image acquisition

Images were acquired from a 3 Tesla Siemens Magnetom Prisma MRI scanner with a 64-channel head coil at the University of Kentucky’s Magnetic Resonance Imaging and Spectroscopy Center (MRISC). Data from four sequences were collected in the following order: (1) a 3D multi-echo, T1-weighted magnetization prepared rapid gradient echo (T1) sequence; (2) a 3D fluid-attenuated inversion recovery (FLAIR) sequence; (3) a spin-echo, echo-planar multi-shell diffusion-weighted sequence, and (4) a spin-echo, echo-planar diffusion-weighted sequence with reverse phase-encoding direction from the main diffusion sequence. Data from several other sequences pertaining to unrelated scientific questions were also collected during the session and are not further described here.

The T1 scan had four echoes [first echo time (TE) = 1.69 ms, echo spacing = 1.86 ms], and covered the entire brain [256 × 256 × 176 mm acquisition matrix (176 slices), repetition time (TR) = 2,530 ms, 1 mm isotropic voxels, flip angle = 7°, scan duration = 5.88 min]. The 3D FLAIR sequence covered the entire brain (256 × 256 × 176 mm acquisition matrix, TR = 5,000 ms, TE = 38 ms, 1 mm isotropic voxels, inversion time = 1800 ms, scan duration = 6.45 min). The main multi-shell diffusion sequence was acquired with 126 separate diffusion directions [232 × 232 × 162 mm acquisition matrix (81 slices), TR = 3,400 ms, TE = 71 ms, 2 mm isotropic voxels, posterior-to-anterior phase encoding direction, multislice acceleration factor = 3, phase partial Fourier = 6/8, and scan duration = 7.45 min] divided between 4 b-values [0 s/mm^2^ (12 directions), 500 s/mm^2^ (6 directions), 1,000 s/mm^2^ (48 directions), and 2000 s/mm^2^ (60 directions)]. The short (28-s) diffusion sequence was collected using the same parameters as the main diffusion sequence but used the reverse-phase encoding direction (anterior-to-posterior phase encoding direction) and 2 b-values (0 and 2000 s/mm^2^). The non-diffusion weighted (b = 0 s/mm^2^) images were used to correct for susceptibility-induced distortions as recommended by FSL’s topup (35).

### 2.3 T1 segmentation

The four echoes from the multi-echo T1 scan were averaged into a root mean square (RMS) image, as described previously (36). The RMS T1 images were then examined visually to ensure no participants had significant brain abnormalities, motion artifacts, or poor contrast that could interfere with segmentation accuracy. All T1 images passed quality control criteria and were automatically segmented into gray matter, white matter and cerebrospinal fluid (CSF) using the recon-all option in FreeSurfer 6.0 (37). Estimated intracranial volume (eICV, mm^3^) was used as a covariate in subsequent analyses.

### 2.4 White matter hyperintensity analyses

White matter hyperintensity volume (WMH volume) was computed using a validated 4-tissue segmentation method (38, 39) used in the Alzheimer’s Disease Neuroimaging Initiative (ADNI) analysis pipeline and the MarkVCID consortium (28).^2^ First, participant’s FLAIR images were registered to their T1 image (RMS image; see *section 2.3*) using FLIRT from the FMRIB Software Library version 6.0.1 [FSL; (40)]. FLAIR images were then corrected for inhomogeneities using local histogram normalization (41) and were non-linearly aligned to a minimal deformation template (39).

FLAIR images were then segmented in template space using Bayesian probability based on histogram fitting and prior probability maps. Voxels segmented as WMHs must also have exceeded 3.5 SDs above the mean WM signal intensity. WMHs were visually examined to ensure quality segmentation and volume estimation. Manual editing was accomplished by labeling false positive FLAIR hyperintensities as background, typically in the septum pellucidum and other membranes surrounding the ventricular areas. The segmented WMH volumes were then back-transformed to native space where the total volume for each participant was reported in cubic millimeters. Finally, each participant’s WMH volume was log transformed, as WMH volume was non-normally distributed in our sample.

### 2.5 Free water and peak width of skeletonized mean diffusivity analysis

#### 2.5.1 Diffusion-weighted imaging preprocessing

Pre-processing and processing for diffusion MRI data has been described in our previous work (42). Briefly, each participant’s diffusion data was corrected for susceptibility induced distortions using a reverse phase-encoded scan in FSL’s topup (35), skull-stripped with BET (43), and corrected for eddy currents and participant motion with eddy (44). All diffusion MRI data was examined visually to ensure quality. No participants had 2 mm or greater average head motion across volumes as assessed using eddy QC tools [eddyqc tools: QUality Assessment for DMRI (QUAD) and Study-wise QUality Assessment for DMRI (SQUAD)], and therefore no participants were excluded for excessive motion.

#### 2.5.2 Diffusion-tensor processing

Fractional anisotropy (FA) maps were calculated using FSL’s DTIFIT. This function computes the diffusion tensor model and eigenvalues within each voxel from each participant’s preprocessed diffusion MRI data.

#### 2.5.3 Free water processing

Free water (FW) maps were computed for each participant using the FW kit, developed and validated through the MarkVCID consortium (28), which in this study was adapted for multi-shell diffusion data. FW maps were calculated for each participant using a two-compartment model of the multi-shell Free Water Diffusion Tensor Imaging algorithm (45) from the open-source software package Diffusion Imaging in Python [DIPY; (46)]. FW maps were then transformed into standard space (standard FSL template FMRIB 1-mm FA template) using parameters calculated by registering the participant’s FA map to the same standard space. White matter throughout the brain (global white matter) is then defined using the FMRIB 1-mm FA template after applying a 0.3 threshold to reduce cerebrospinal fluid partial volume contamination. This thresholded template is then used an as ROI mask. The mean value of all voxels in the FW map within this ROI are then extracted to produce the mean free water value for each participant.

#### 2.5.4 Peak width of skeletonized mean diffusivity processing

Peak width of skeletonized mean diffusivity (PSMD) (47) was computed for each participant using the PSMD kit developed and validated through the MarkVCID consortium (28). First, each participant’s FA map was registered to a standard FSL template (FMRIB 1-mm FA). FSL’s Tract-Based Spatial Statistics (TBSS) pipeline was then used to produce a group FA skeleton, with each participants’ FA data projected onto the skeleton. A threshold of 0.2 was then used exclude non-WM voxels. Each participant’s MD map is then projected onto the thresholded FA skeleton using the FA-derived projection parameters, and further thresholded with a template skeleton mask to reduce CSF volume contamination. Finally, PSMD is calculated as the difference between the 95^th^ and 5^th^ percentiles in MD values within the skeleton.

### 2.6 Calculation of CRIq scores

The CRIq was administered according to the published instructions (32). Broadly, the CRIq asks participants about frequency and the number of years engaged in several leisure activities throughout the lifespan (CRIq-Leisure Time), the number of years worked in various occupations throughout the lifespan (CRIq-Working Activity), and the number of years of early life education (CRIq-Education). Scores from each of the three subscales are then calculated based on residuals from linear regression models (32). The total CRIq score is then calculated as the average of the three subscores (32).

### 2.7 Nutrition data acquisition and analysis

The nutrition factor used in this study (which represents nutrients commonly found in fish, healthy oils and nuts: FON factor) was identified as part of our previous work exploring the role of dietary factors on brain iron levels in older adults (48). Three nutrition factors were identified in that work, as described below. In the present work, we focused on dietary factor 2 (FON factor) as a potential reserve variable against cSVD because it constitutes a core component of the Mediterranean diet (49) and was the only factor that was correlated with cognitive performance in our previous work (48).

Participants completed an online version of the “Newly Developed Antioxidant Nutrient Questionnaire” (NDANQ) (34), where they self-reported the quantity they consumed per day of 92 food items and 15 over-the-counter supplements/multivitamins during the preceding month (48). Survey data was processed using in-house developed software (48) to convert foods consumed per day into milligrams (mg) of nutrients per day on a total of 122 nutrients. This conversion was done automatically using the United States Department of Agriculture (USDA) Research Service databases: the National Nutrient Database for Standard Reference,^3^ the flavonoid values for USDA survey foods and beverages (50),^4^ and the food and nutrient database for dietary studies.^5^ A literature review was then used to narrow the nutrient list down to only a subset that can cross the blood brain barrier and either chelate brain iron or reduce oxidative stress. Following this criteria the nutrient subset was narrowed to vitamin C, vitamin E, quercetin, lysine, epigallocatechin 3-gallate, *β*-carotene, β-cryptoxanthin, docosahexaenoic acid omega-3 (DHA), and omega-6 polyunsaturated fatty acids (PUFAs) (51).

Nutrients that co-varied were grouped into largely independent nutrition factors (48) using exploratory factor analysis in SPSS 27 (IBM, Chicago, IL, United States). The analysis used the nutrients identified in the previous paragraph as inputs, with principal components as the extraction method under the assumption that factors may not be independent (48). Factor scores were created for the nutrition factors using the regression method (52). Ultimately, 3 dietary factors were identified with this analysis. Vitamin C, quercetin, β-carotene, and β-cryptoxanthin loaded onto dietary factor 1, vitamin E, lysine, docosahexaenoic acid omega-3, and omega-6 polyunsaturated fatty acids loaded onto dietary factor 2, while epigallocatechin 3-gallate loaded onto dietary factor 3. In terms of food groups, dietary factor 1 largely represents nutrients derived from common vegetables and fruits (VF factor), dietary factor 2 largely represents nutrients derived from fish, (healthy) oils, and nuts (FON factor), while dietary factor 3 represents epigallocatechin 3-gallate, which is found in many herbs and tea (48).

### 2.8 Statistical analyses

We first explored whether reduced cSVD would be associated with better cognition (brain reserve) and/or whether FON dietary intake may interact with cSVD when predicting cognition (cognitive reserve). Three multivariate linear regression models were conducted to test FON factor, VCID biomarkers, and the interaction between FON factor and VCID biomarkers, as predictors of MoCA scores. Each model used a different VCID biomarker (WMH volume, FW, or PSMD) as a predictor. All models were corrected for age (years), sex, education (years), and estimated intracranial volume (eICV; mm^3^).

We then explored whether FON dietary intake might help preserve brain health relative to chronological age (as a brain maintenance variable). Three additional multivariate linear regression models were conducted to test FON factor, age, and the interaction between FON factor and age, as predictors of VCID biomarkers. Each model used a different VCID biomarker (WMH volume, FW, PSMD) as the dependent variable. Sex, education (years) and estimated intracranial volume (eICV; mm^3^) were included as covariates in each model.

Finally, we assessed the potential specificity of FON dietary intake as a reserve factor by substituting in a more traditional reserve proxy in all models. All six multivariate linear regression models described above were re-run replacing FON factor with the CRIq (32). Covariates remained the same as they were in the original models, except that education was removed as a covariate due to its similarity to the CRIq.

Statistical analyses were conducted using SPSS 28 (IBM, Chicago, IL, United States). Results were considered statistically significant at *p* < 0.05 using a false discovery rate (FDR) approach (53). Both MoCA scores and WMH volume were not normally distributed and were therefore log transformed. Statistical outliers were defined as values greater than 3.29 standard deviations from the group mean and were excluded from relevant analyses. Error residuals in all linear regression models were examined for the assumption of normality. Multivariate linear regression models with moderation were conducted through the PROCESS macro (version 4.0) in SPSS (54). Moderator variables were mean centered. Significant interactions were probed using the pick-a-point approach, which uses all the data (no grouping) to demonstrate moderation effects at 16% (low), 50% (medium), and 84% (high) of the moderator value (which corresponds to the mean value + − 1 SD) (54). VCID biomarkers were considered to be the independent variable while reserve variables (FON factor, CRIq scores) were considered to be the moderator when probing interactions for cognitive reserve. Statistics for significance (*p*-values) in moderation probing indicate whether the slope of the line at the chosen moderation point (low, medium, or high) is significantly different from zero, and are included to fully describe our results.

## 3 Results

Participant demographics are summarized in Table 1. One participant was an outlier for MoCA scores, one participant was an outlier for PSMD, and one participant was an outlier for WMH volume. Data from these participants were excluded from the analyses in which they were outliers. Error residuals followed an approximate normal distribution in all regression models. All results reported below pertain to findings after correction for multiple comparisons, although uncorrected *p*-values are also reported for transparency.

**Table 1:** Group demographics, biomarker metrics, and Montreal cognitive assessment (MoCA) scores.

	Mean (S.D.)	*N*
Age (Years)	70.1 (5.9)	71
Sex Ratio (F:M)	43:28	71
Education (Years)	16.7 (2.3)	71
WMH volume	4.71 (5.44)	70
Free water	0.258 (0.015)	71
PSMD	0.27 (0.050)	70
CRIq score	134.6 (17.8)	71
MoCA	26.6 (2.5)	70

CRIq, Cognitive Reserve Index questionnaire; F:M, female to male; standard deviation; MoCA, Montreal Cognitive Assessment; PSMD, Peak Skeletonized Mean Diffusivity; S.D., standard deviation; WMH, White Matter Hyperintensity. The table lists the female/male ratio and mean (± sd) age (years), education (years), white matter hyperintensity volume (cm^3^), free water (fraction), peak skeletonized mean diffusivity (mm^2^/s x 10^−3^), total Cognitive Reserve Index questionnaire (CRIq) scores, and MoCA scores for the full sample.

**Figure 1:**
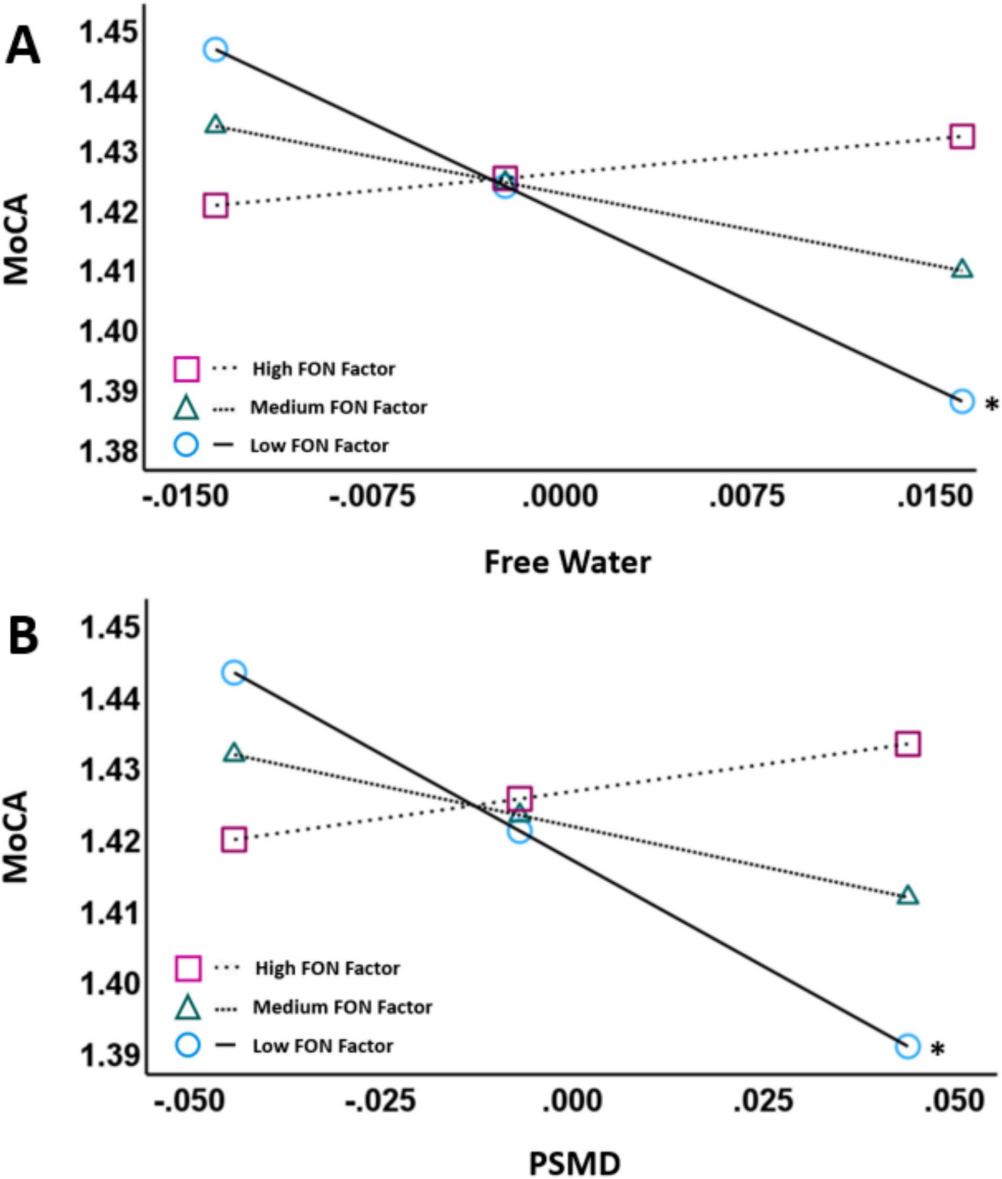
Partial regression plots showing FW **(A)** and PSMD **(B)** against MoCA scores. The pick-a-point approach was used to determine whether the slope of the regression line between VCID biomarkers (FW, PSMD; residualized; X-axis) and MoCA scores (log-transformed; Y-axis) were significantly different from zero at 3 different moderator values [High FON factor, Medium FON factor, and Low FON factor]. Both plots control for age, sex, education and ICV. The moderator variable was mean centered. The figures illustrate that there is a negative association between the VCID biomarkers and MoCA scores at low FON factor intake values (*p* < 0.01) but not at medium or high FON factor intake values. Abbreviations: MoCA, Montreal Cognitive Assessment; PSMD, Peak Skeletonized Mean Diffusivity. *p < 0.05.

We first explored associations between VCID biomarkers (WMH volume, FW or PSMD) and MoCA scores. There were trends but no main effects of WMH volume (uncorrected *p* = 0.091), FW (uncorrected *p* = 0.053) or PSMD (uncorrected *p* = 0.070) on MoCA scores. However, there was an interaction between the FON factor and both FW (uncorrected *p* = 0.009) and PSMD (uncorrected *p* = 0.008; Table 2; Figure 1) when predicting MoCA scores. There was no interaction between FON factor and WMH volume (uncorrected *p* = 0.137) when predicting MoCA scores.

**Table 2:** Associations between VCID biomarkers and MoCA scores using the FON factor as the reserve variable.

Predictor	Unstandardized *β*	95% CI for *β*	*p* value
Model 1			
Age	−0.0009	−0.0030 – 0.0013	0.427
Sex	−0.0309	−0.0602 – −0.0015	0.040*
Education	0.0014	−0.0034 – 0.0062	0.567
Estimated intracranial volume	0.0000	0.0000–0.0001	0.346
FON factor	0.0040	−0.0067 – 0.0146	0.457
WMH volume	−0.0218	−0.0471 – 0.0036	0.091
FON factor x WMH volume	0.0191	−0.0060 – 0.0442	0.137
Model 2			
Age	−0.0007	−0.0027 – 0.0014	0.515
Sex	−0.0310	−0.0578 – −0.0042	0.024*
Education	0.0016	−0.0029 – 0.0061	0.485
Estimated intracranial volume	0.0000	0.0000–0.0001	0.261
FON factor	0.0033	−0.0065 – 0.0132	0.503
Free Water	−0.8128	−1.6379 –0.0123	0.053
FON factor x Free water	1.2239	0.3152–2.1325	0.009*
Model 3			
Age	−0.0008	−0.0029 – 0.0013	0.447
Sex	−0.0278	−0.0552 – −0.0003	0.047*
Education	0.0023	−0.0024 – 0.0069	0.337
Estimated intracranial volume	0.0000	0.0000–0.0001	0.405
FON factor	0.0050	−0.0052 – 0.0152	0.331
PSMD	−0.2274	−0.4743 – 0.0194	0.070
FON factor x PSMD	0.3885	0.1044–0.6727	0.008*

CI, confidence interval; FON, fish oils and nuts; MoCA, Montreal Cognitive Assessment; PSMD, Peak Skeletonized Mean Diffusivity; WMH, White Matter Hyperintensity. #*p* < 0.05 before, *p < 0.05 after correction for multiple comparisons. The table displays the unstandardized *β* estimates, the 95% confidence intervals (CI) for *β* estimates, and the *p* values for each predictor in the linear regression models. A different VCID biomarker is explored in each model.

We decomposed the significant interactions between VCID biomarkers (FW and PSMD) and the FON factor on MoCA scores using the pick-a-point approach as described by Hayes (54). This approach determines if the slope of the partial regression plot line between VCID biomarkers and MoCA scores is significantly different from zero at three different values of the moderator [FON factor; 16% (low), 50% (medium), 84% (high)] and provides better characterization of the observed interaction than the interaction terms alone (54). Using this approach, there was a significant negative association between FW and MoCA at low values of the FON factor (Unstandardized *β* = −1.9627, 95% CI = −3.0440 – −0.8813, *p* < 0.001) but not between FW and MoCA at medium values of the FON factor (*p* = 0.055) or high values of the FON factor (*p* = 0.562; Figure 1A). Similarly, there was a negative association between PSMD and MoCA at low values of the FON factor (Unstandardized β = −0.5953, 95% CI = −0.9742 – −0.2165, *p* = 0.003), but not at medium (*p* = 0.071) or high (*p* = 0.399) values (Figure 1B).

We then explored potential main effects of age on VCID biomarkers (WMH volume, FW, PSMD) and whether those effects were moderated by the FON factor. There was a main effect of age on each of the VCID biomarkers [WMH volume (uncorrected *p* = 0.002), FW (uncorrected *p* < 0.001), and PSMD (uncorrected *p* < 0.001)]. However, the FON factor did not moderate the association between age and VCID biomarkers in any of the models (Table 3).

**Table 3:** Associations between age and vascular biomarkers using FON factor as the reserve variable.

Predictor	Unstandardized *β*	95% CI for *β*	*p* value
Model 1: WMH volume is DV			
Age	0.0296	0.0111–0.0481	0.002*
Sex	−0.1802	−0.4647 – 0.1042	0.210
Education	0.0010	−0.0464 – 0.0484	0.967
Estimated intracranial volume	0.0011	0.0003–0.0018	0.007*
FON factor	−0.0290	−0.1373 – 0.0794	0.595
Age x FON factor	0.0074	−0.0127 – 0.0276	0.462
Model 2: Free water is DV			
Age	0.0010	0.0005–0.0016	< 0.001*
Sex	0.0009	−0.0072 – 0.0090	0.829
Education	−0.0002	−0.0015 – 0.0012	0.789
Estimated intracranial volume	0.0000	0.0000–0.0001	0.005*
FON factor	0.0001	−0.0030 – 0.0032	0.937
Age x FON factor	0.0003	−0.0003 – 0.0009	0.311
Model 3: PSMD is DV			
Age	0.0040	0.0022–0.0058	< 0.001*
Sex	−0.0008	−0.0289 – 0.0272	0.952
Education	−0.0001	−0.0049 – 0.0047	0.967
Estimated intracranial volume	0.0001	0.0000–0.0002	0.018*
FON factor	−0.0005	−0.0113 – 0.0102	0.921
Age x FON factor	0.0005	−0.0015 – 0.0025	0.610

CI, confidence interval; DV, Dependent Variable; FON, fish oils and nuts; PSMD, Peak Skeletonized Mean Diffusivity; WMH, White Matter Hyperintensity. #*p* < 0.05 before, *p < 0.05 after correction for multiple comparisons. The table displays the unstandardized β estimates, the 95% confidence intervals (CI) for β estimates, and the *p* values for each predictor in the linear regression models. In each model, there is a different vascular biomarker as the dependent variable.

When total CRIq scores were used as our reserve variable, there were no main effects of WMH volume (uncorrected *p* = 0.695), FW (uncorrected *p* = 0.047), or PSMD (uncorrected *p* = 0.251) on MoCA scores (Table 4). There were no interactions between total CRIq scores and WMH volume (uncorrected *p* = 0.436), FW (uncorrected *p* = 0.311), or PSMD (uncorrected *p* = 0.695) when predicting MoCA scores. In the three models exploring brain maintenance, CRIq scores also did not moderate the association between age and VCID biomarkers (Table 5). There were no significant main effects or interactions when replacing total CRIq scores with scores specific to any of its subscales (CRIq-Education, CRIq-Working Activity, or CRIq-Leisure Time) in any models.

**Table 4:** Associations between VCID biomarkers and MoCA scores using total CRIq scores as the reserve variable.

Predictor	Unstandardized *β*	95% CI for *β*	*p* value
Model 1			
Age	−0.0009	−0.0030 – 0.0014	0.406
Sex	−0.0360	−0.0646 – −0.0069	0.014*
Estimated intracranial volume	0.0000	0.0000–0.0001	0.228
CRIq scores	0.0005	−0.0003 – 0.0013	0.231
WMH volume	−0.0242	−0.0506 – 0.0023	0.069
CRIq x WMH volume	−0.0007	−0.0023 – 0.0009	0.436
Model 2			
Age	−0.0006	−0.0029 – 0.0016	0.589
Sex	−0.0262	−0.0531 – 0.0007	0.056
Estimated intracranial volume	0.0001	0.0000–0.0001	0.196
CRIq scores	0.0003	−0.0004 – 0.0009	0.433
Free water	−0.8763	−1.7390 – 0.0135	0.047#
CRIq x Free water	−0.0203	−0.0601 – 0.0194	0.311
Model 3			
Age	−0.0013	−0.0036 – 0.0010	0.279
Sex	−0.0259	−0.0536 – 0.0018	0.067
Estimated intracranial volume	0.0000	0.0000–0.0001	0.417
CRIq scores	0.0005	−0.0003 – 0.0012	0.200
PSMD	−0.1604	−0.4373 – 0.1164	0.251
CRIq x PSMD	−0.0028	−0.0170 – 0.0114	0.695

CI, confidence interval; CRIq, Cognitive Reserve Index Questionnaire; MoCA, Montreal Cognitive Assessment; PSMD, Peak Skeletonized Mean Diffusivity; WMH, White Matter Hyperintensity. #*p* < 0.05 before, *p < 0.05 after correction for multiple comparisons. The table displays the unstandardized β estimates, the 95% confidence intervals (CI) for β estimates, and the *p* values for each predictor in the linear regression models. A different VCID biomarker is explored in each model.

**Table 5:** Associations between age and vascular biomarkers using CRIq scores as the reserve variable.

Predictor	Unstandardized *β*	95% CI for *β*	*p* value
Model 1: WMH volume is DV			
Age	0.0296	0.0108–0.0484	0.003*
Sex	−0.1891	−0.4690 – 0.0908	0.182
Estimated intracranial volume	0.0011	0.0004–0.0019	0.004*
CRIq scores	−0.0010	−0.0075 – 0.0055	0.757
Age x CRIq scores	−0.0004	−0.0016 – 0.0008	0.533
Model 2: Free water is DV			
Age	0.0011	0.0006–0.0017	0.001*
Sex	0.0008	−0.0070 – 0.0086	0.842
Estimated intracranial volume	0.0000	0.0000–0.0001	0.001*
CRIq scores	−0.0002	−0.0003 – 0.0000	0.082
Age x CRIq scores	0.0000	0.0000–0.0000	0.560
Model 3: PSMD is DV			
Age	0.0043	0.0024–0.0061	< 0.001*
Sex	0.0016	−0.0288 – 0.0256	0.907
Estimated intracranial volume	0.0001	0.0000–0.0002	0.008*
CRIq scores	−0.0003	−0.0010 – 0.0003	0.285
Age x CRIq scores	0.0000	−0.0001 – 0.0001	0.612

CI, confidence interval; CRIq, Cognitive Reserve Index questionnaire; DV, Dependent Variable; PSMD, Peak Skeletonized Mean Diffusivity; WMH, White Matter Hyperintensity. #*p* < 0.05 before, *p < 0.05 after correction for multiple comparisons. The table displays the unstandardized β estimates, the 95% confidence intervals (CI) for β estimates, and the *p* values for each predictor in the linear regression models. In each model, there is a different vascular biomarker as the dependent variable.

As noted above, our analyses focused on the FON factor as it was the only dietary factor that was correlated with cognitive performance in our previous work (48). However, supplementary analyses were conducted to investigate potential moderation effects between VCID biomarkers and nutrients derived from common vegetables and fruits (VF factor; section 2.7) on MoCA scores, due to fruits and vegetables also being a component of the Mediterranean diet (20, 24, 25). Results indicated that, as with FON factor, there was a significant interaction between the VF factor and FW when predicting MoCA scores (uncorrected *p* = 0.011; Supplementary Table S1). However, there was no interaction between VF factor and either PSMD (uncorrected *p* = 0.083) or WMH volume (uncorrected *p* = 0.215) when predicting MoCA scores (Supplementary Table S1).

## 4 Discussion

Our results indicated that dietary intake of specific nutrients moderated the relationship between MRI markers of VCID and cognition. Specifically, our findings revealed negative associations between several MRI markers of cSVD (FW, PSMD) and MoCA scores in those with low intake of fish, (healthy) oils, and nuts (FON), but not in those with medium or high FON intake. In contrast, FON intake did not moderate the relationship between age and MRI markers of cSVD. Finally, a different potential reserve variable, scores on the Cognitive Reserve Index questionnaire (CRIq), did not moderate either of these relationships. Our findings suggest that the intake of certain dietary nutrients may build cognitive reserve against cSVD in older adults.

### 4.1 Dietary intake moderates the relationship between diffusion MRI measures of cSVD and cognitive function

Our results demonstrated that the relationships between several established MRI markers of cSVD and cognition were moderated by dietary intake. Specifically, high FW and PSMD values were associated with lower MoCA scores in those with low FON values, but not those with medium or high values. This suggests that healthy dietary intake may promote cognitive reserve against cSVD. Both FW and PSMD are thought to capture subtle cSVD damage associated with VCID (28). Biologically, high FW primarily reflects increased extracellular water content and has been linked to elevated blood pressure and arterial stiffness (arteriosclerosis) (55), while PSMD reflects heterogeneity of the mean diffusivity values across WM tracts (47) and high PSMD has been linked to lower blood flow (56).

In contrast to the moderation described above, the FON factor did not moderate the relationship between WMH volume and cognitive performance. This may be because macrostructural WMHs reflect more advanced cSVD than microstructural diffusion metrics of FW and PSMD. Specifically, WMHs have been linked with gliosis, axonal degeneration, myelin loss, vacuolation and BBB damage/dysfunction (5, 57, 58). This possibility is in-keeping with evidence that FW and PSMD precede WMH development (47, 55), and that diffusion measures are able to detect alterations in normal-appearing WM (59). Other studies have reported that potential reserve variables (education, reading and vocabulary scores, involvement in social activities, physical activity) do not moderate the relationship between WMH volume and cognition (10, 14), although positive findings have also been reported (13, 16, 18). The present results suggest that certain lifestyle variables build cognitive reserve against relatively early/minor cSVD (microscopic diffusion alterations), but may become overwhelmed as cSVD becomes more advanced (i.e., significant WMH burden).

Our results did not find evidence for dietary intake as a measure of brain reserve. While there were trends found in our results, none of the relationships between cSVD biomarkers and MoCA scores reached significance. These findings are somewhat surprising in that high FW and/or PSMD have been associated with poorer cognitive performance in a number of studies, including validation studies as part of the MarkVCID consortium (29, 83). However, FW and PSMD were validated in relation to composite cognitive metrics composed of scores on multiple National Alzheimer’s Coordinating Center (NACC) Uniform Data Set 3 (UDS-3) measures, whereas the MoCA measure used here is less sensitive to performance on specific cognitive domains. Nonetheless, MoCA performance remains an important measure to use in the field of reserve given that it is a sensitive and widely used measure of global cognitive functioning (60). Future studies are needed to determine which cognitive domains may be most preserved by maintaining cerebrovascular health (i.e., brain reserve).

### 4.2 Dietary intake does not moderate the relationship between chronological age and MRI measures of cSVD

Evidence for brain maintenance would come from a finding that a putative reserve variable (here dietary intake) mitigates the negative effects of aging on brain health (here cSVD) (6–8). We did not find supportive evidence for brain maintenance in this study. As expected, all of the cSVD biomarkers in our study (WMH volume, FW, PSMD) were negatively associated with chronological age, consistent with other findings (61–68). However, FON factor intake did not moderate the association between age and any of the VCID biomarkers in our study.

As this is the first study to use the MarkVCID MRI biomarkers of FW and PSMD in an experiment focused on dietary intake as a reserve variable, there does not exist a literature in which we can draw comparisons related to our null findings concerning brain maintenance. However, WMH volume has been used in a number of studies on reserve. In general, our null results concerning brain maintenance are similar to others reporting that neither dietary intake (25, 69) nor education (12) protect against WMHs over time in older adults. In contrast, our previous work has shown that high cardiorespiratory fitness (CRF) does diminish the effects of age on WMH volume, such that age is more strongly associated with WMH volume in older adults with low CRF compared to those with high CRF (11). Together, these findings suggest that reserve factors more directly related to blood flow, such as exercise (11), may be more likely than dietary intake to promote brain maintenance (i.e., resistance to the development of age-related WMHs).

### 4.3 Traditional cognitive reserve measures do not moderate the relationship between diffusion MRI measures of cSVD and cognitive function

Our results further suggest some specificity concerning variables that build cognitive reserve against cSVD. Specifically, unlike healthy dietary intake, scores on the Cognitive Reserve Index questionnaire (CRIq) did not moderate the association between any of the cSVD biomarkers used in this study and MoCA scores. Broadly, the CRIq is comprised of more traditional cognitive reserve variables such as early life education, occupational history, and engagement in recreational activities. Previous work has found that such variables often mitigate the effects of brain pathology on cognitive function (70–73) although null effects have also been reported (74, 75).

Notably, findings related to CRIq as a reserve variable have been reported largely within the context of biomarkers of Alzheimer’s disease (AD). Much less work has been conducted exploring CRIq components as potential reserve variables against biomarkers of cSVD. The few studies that have been conducted report fairly mixed findings (10, 14, 15, 17). Overall, the evidence that more traditional cognitive reserve variables actually provide reserve appears less consistent in cSVD than AD.

### 4.4 Dietary intake as a cognitive reserve variable: some potential underlying mechanisms

The FON factor we used is comprised of the nutrients vitamin E, lysine, DHA, and omega-6 polyunsaturated fatty acids (PUFAs), which are commonly found in the Mediterranean Diet (49). PUFAs [which include DHA (which is an omega-3 PUFA) and omega-6 PUFAs] in particular are well documented in their positive relationship with cardiovascular function (76–79) through a variety of factors including the lowering of blood pressure (76, 78, 79) and plasma cholesterol levels (77–79), increasing general endothelial function (76, 79), and the reduction of atherosclerosis (76, 79).

Recent evidence also suggests omega-3 PUFAs may protect blood–brain barrier (BBB) integrity and alleviate glymphatic dysfunction (78) which are critical to cerebrovascular health. Furthermore, all nutrients included in the FON factor have antioxidant properties (48), which may counteract damage to the endothelium and BBB caused by cSVD-induced oxidative stress (80, 81). The consumption of these nutrients in older adults may then help to counteract (and thereby provide some reserve against) cSVD disease processes captured by FW and PSMD, which would help to preserve cognitive function across the numerous domains associated with the Mediterranean diet (26, 27).

Nevertheless, we do not conclude that nutrients comprising the FON factor are the only ones that may build cognitive reserve against cSVD. In particular, fruits and vegetables (VF factor) represent a component of the Mediterranean diet known to be beneficial to cerebrovascular health (20, 24, 25). In our supplementary analysis, we also found that VF factor moderated the association between FW and MoCA scores, suggesting that the intake of vegetables and fruit may contribute some reserve against cSVD. Additional studies will be required to determine the separate and possibly joint effects of different dietary factors on cognitive reserve. Future work using functional neuroimaging should also assess if the cognitive reserve effects associated with healthy dietary intake result from better maintenance of network connectivity, or perhaps reorganization of functional brain networks in aging.

### 4.5 Strengths and limitations

Strengths of the study include the use of multiple, validated biomarkers of VCID (WMH volume, FW, PSMD) (28–30, 82) developed by a multicenter consortium,^6^ in the context of a study on reserve. Future work is needed to establish standardized VCID biomarker cutoff thresholds for group comparisons as is done with AD using PET amyloid positivity/negativity. Further, FON factor itself has been previously validated and demonstrated to be strongly associated with cognitive performance using composite measures (48). Finally, our use of statistical moderation models to test for potential reserve is the “gold standard” in the field (7).

One primary limitation of our study is the cross-sectional design which limits interpretation and prevents causal inferences. Brain maintenance in particular is best investigated longitudinally (6, 7). In addition, our measure of nutrient intake (NDANQ) and responses on the CRIq were based on self-report. Future research investigating nutrient patterns as a potential reserve proxy in VCID should ideally use fluid-based nutrient markers. The MoCA, while clinically relevant, is also not a comprehensive measure of cognitive function. Finally, our highly educated, primarily white sample may limit the external validity of our results to more diverse cohorts. Nevertheless, this work is one of the first to investigate dietary patterns as a measure of reserve against cSVD, and the results may aid in the development of longitudinal studies.

## 5 Conclusion

In conclusion, our results suggest that regular intake of nutrients commonly found in fish, healthy oils and nuts may contribute to cognitive reserve against cSVD. This effect appeared relatively specific to healthy dietary intake as a composite score of early life education, occupational history, and engagement in recreational activities did not protect cognition from cSVD in this study. Future research should attempt to replicate these findings longitudinally and expand the types of reserve variables explored with respect to cSVD in aging populations.

## Data Availability

The raw data supporting the conclusions of this article will be made available by the authors, without undue reservation.

## Footnotes

^1^ https://mocacognition.com/faq/

^2^ https://markvcid.partners.org

^3^release 28; May 2016; https://www.ars.usda.gov/northeast-area/beltsville-md-bhnrc/beltsville-human-nutrition-research-center/methods-and-application-of-food-composition-laboratory/mafcl-site-pages/sr11-sr28/

^4^ https://www.ars.usda.gov/northeast-area/beltsville-md-bhnrc/beltsville-human-nutrition-research-center/food-surveys-research-group/docs/fndds-flavonoid-database/

^5^ https://www.ars.usda.gov/northeast-area/beltsville-md-bhnrc/beltsville-human-nutrition-research-center/food-surveys-research-group/docs/fndds-download-databases/

^6^MarkVCID: https://markvcid.partners.org/
