# Supplementary material for: Healthy dietary intake diminishes the effect of cerebral small vessel disease on cognitive performance in older adults
Source: Front Neurol. 2025 Mar 6;16:1508148. doi: 10.3389/fneur.2025.1508148 (PMC11925079; doi:10.3389/fneur.2025.1508148)
Supplement: Supplementary file 1 [file Table_1.docx]

**Table S1.** Associations between VCID biomarkers and MoCA scores using the VF factor as the reserve variable. The table displays the unstandardized β estimates, the 95% confidence intervals (CI) for β estimates, and the *p* values for each predictor in the linear regression models. A different VCID biomarker is explored in each model.

| Table S1 |  |  |  |
| --- | --- | --- | --- |
| Predictor | **Unstandardized β** | **95% CI for β** | ***p* value** |
| Model 1 |  |  |  |
| Age | -0.0010 | -0.0032 – 0.0011 | 0.345 |
| Sex | -0.0322 | -0.0610 – -0.0035 | 0.029* |
| Education | 0.0015 | -0.0033 – 0.0064 | 0.530 |
| Estimated Intracranial Volume | 0.0001 | 0.0000 – 0.0001 | 0.193 |
| VF factor | 0.0078 | -0.0034 – 0.0190 | 0.169 |
| WMH Volume | -0.0247 | -0.0498 – 0.0003 | 0.053 |
| VF factor x WMH Volume | 0.0172 | -0.0103 – 0.0447 | 0.215 |
| Model 2 |  |  |  |
| Age | -0.0007 | -0.0028 – 0.0013 | 0.464 |
| Sex | -0.0337 | -0.0604 – -0.0071 | 0.014* |
| Education | 0.0017 | -0.0028 – 0.0062 | 0.455 |
| Estimated Intracranial Volume | 0.0001 | 0.0000 – 0.0002 | 0.054 |
| VF factor | 0.0097 | -0.0010 – 0.0205 | 0.075 |
| Free Water | -0.9847 | -1.7947 – -0.1746 | 0.018# |
| VF factor x Free Water | 1.1265 | 0.2727 – 1.9803 | 0.011* |
| Model 3 |  |  |  |
| Age | -0.0010 | -0.0031 – 0.0012 | 0.360 |
| Sex | -0.0291 | -0.0571 – -0.0011 | 0.042* |
| Education | 0.0021 | -0.0027 – 0.0069 | 0.390 |
| Estimated Intracranial Volume | 0.0001 | 0.0000 – 0.0001 | 0.190 |
| VF factor | 0.0094 | -0.0022 – 0.0210 | 0.110 |
| PSMD | -0.2485 | -0.5075 – 0.0105 | 0.060 |
| VF factor x PSMD | 0.2392 | -0.0324 – 0.5107 | 0.083 |
|  |  |  |  |

Abbreviations: MoCA, Montreal Cognitive Assessment; CI, confidence interval; FON, fish oils and nuts; WMH, White Matter Hyperintensity; PSMD, Peak Skeletonized Mean Diffusivity. #*p* < 0.05 before, **p* < 0.05 after correction for multiple comparisons.
